# Memory Aging Phenotypes Among Older Cancer Survivors: A Latent Growth Analysis of the Health and Retirement Study

**DOI:** 10.1093/geroni/igab046.2580

**Published:** 2021-12-17

**Authors:** Ashly Westrick, Kenneth Langa, Lindsay Kobayashi

**Affiliations:** University of Michigan, Ann Arbor, Michigan, United States

## Abstract

While cancer survivors experience many long-term health effects, there is limited evidence on the potentially heterogeneous memory aging of older cancer survivors. We identified memory aging phenotypes of older US cancer survivors, and determined sociodemographic and health-related predictors of membership. Data were from 2,755 survivors aged ≥50 in the U.S. Health and Retirement Study (1998 – 2016). Self-reported first incident cancer diagnosis (except non-melanoma skin cancer) and memory (composite immediate and delayed word-list recall score, combined with proxy-reported cognition) were assessed at biennial interviews. Memory aging phenotypes were identified using latent growth curve (LGC) models, with baseline being time of cancer diagnosis. Logistic regression evaluated predictors of group membership. 5 distinct memory aging groups were identified: low memory (n=165, 6.16%); medium-low memory (n=459, 17.1%); medium-high memory (n=733, 27.4%); high memory (n=750, 28.0%); and very high memory (n=571, 21.3%). The low memory group received less chemotherapy compared to the other groups (20.0% vs. 25.5%, 31.7%, 36.8%, 41.5%%, respectively), and had the shortest mean survival time after diagnosis (1.08 vs 2.10, 2.76, 3.37, 4.31 years, respectively). Older age at diagnosis (OR: 1.71, 95%CI: 1.61-1.82), being male (OR: 4.10, 95%CI: 2.82-6.51), having a history of stroke (OR: 4.62, 95%CI: 2.57-8.30) and depression prior to diagnosis (OR: 1.19, 95%CI: 1.05-1.34) were independently associated with being in the low memory group vs. the medium-high memory group. We identified distinct memory aging phenotypes among older cancer survivors. Further research should evaluate the influence of pre-cancer memory and how these phenotypes differ from the general population.

